# The 200 most influential publications in migraine research: a bibliometric mapping of the intellectual landscape

**DOI:** 10.3389/fneur.2025.1711571

**Published:** 2025-12-01

**Authors:** Qianxiu Chen, Shijie Wei, Guoliang Jiang, Xiaowei Hu, Lili Zhang, Pengcheng Li, Jing Han

**Affiliations:** 1 School of Acupuncture and Tuina, Shandong University of Traditional Chinese Medicine, Jinan, China; 2 Institute of Acupuncture and Moxibustion, Shandong University of Traditional Chinese Medicine, Jinan, China; 3 Department of Acupuncture, Affiliated Hospital of Shandong University of Traditional Chinese Medicine, Jinan, China

**Keywords:** migraine, bibliometrics, VOSviewer, Scimago Graphica, CiteSpace, Bibliometrix

## Abstract

**Background:**

Migraine is a prevalent and highly disabling neurological disorder. A systematic evaluation of its foundational literature is essential for advancing management strategies. This study employed bibliometric methods to trace the historical trajectory of migraine research and identify principal developmental trends.

**Methods:**

The Web of Science and PubMed database was searched to identify the 200 most highly cited publications, filtered by date, language, and document type. Comprehensive analyses and visualizations were conducted using bibliometrix (R), VOSviewer, Scimago Graphica, CiteSpace, and Microsoft Excel.

**Results:**

The selected publications appeared in 45 journals across 45 countries, authored by 4,409 researchers from 1,592 institutions. Together, these works incorporated 860 keywords and cited 7,995 references. Neurology, Cephalalgia, and Headache were the leading journals, while Lipton RB and Goadsby PJ were the most influential authors. The United States led in publication volume, with Albert Einstein College of Medicine among the top institutions. Ten landmark papers were highlighted, and their contributions, along with those of the broader corpus, were systematically reviewed. Key research hotspots were also delineated.

**Conclusion:**

Current high-impact research emphasizes treatment, therapeutic targets, genetics, the trigeminovascular system, pain modulation, neuroimmunology, aura, and comorbidities. Recent studies confirm sustained interest in these areas, with increasing focus on the brain–gut axis. Future directions include deeper investigation of migraine pathophysiology, especially the calcitonin gene-related peptide system and episodic migraine subtypes. Rigorous methodologies and emerging technologies will enhance evidence-based evaluations of long-term safety and efficacy, while multidimensional assessments of pharmacological and non-pharmacological therapies are expected to expand.

## 1 Introduction

Migraine is a common and highly disabling neurologic disorder affecting over 15% of the global population, imposing a substantial burden worldwide (1, 2). Over the years, the academic community has continuously conducted extensive clinical and mechanistic studies, leading to significant advancements in the understanding of migraine pathophysiology. As a method for the comprehensive analysis of publications within a specific field, bibliometrics enables researchers to systematically assess the structure and evolution of the research landscape (3, 4). The Web of Science (WOS) database is widely recognized for the high quality of its indexed publications and its comprehensive citation data (5). PubMed, as the primary database for biomedical and life sciences literature, provides extensive coverage of clinical and medical research (6). Therefore, to better elucidate the research priorities in this field, we employed both the WOS and PubMed database to conduct a bibliometric and visualization-based analysis of the 200 most highly cited publications on migraine. We systematically summarized relevant information on publications, journals, authors, countries, institutions, keywords, and cited references. Through this analysis results, we elucidate the overall landscape, identified major areas of focus, and highlighted emerging trends in migraine research. Subsequently, the most recently published literature was compared with our findings to assess the current level of attention given to the identified research priorities. This study is expected to contribute to a deeper understanding of key research hotspots within the field and to provide valuable insights for improving future clinical diagnosis and treatment strategies.

## 2 Materials and methods

### 2.1 Data collection

Literature retrieval was conducted using the WOS Science Citation Index Expanded (SCI-E) database^1^ and PubMed.^2^ The detailed search strategy is provided in Supplementary Data 1. The search included only English-language articles and reviews published through December 31, 2024. A comprehensive search strategy was employed across both databases. In WOS, 50,007 publications were identified, while PubMed yielded 48,296 publications. After removing duplicates between databases using DOI and title-author matching, 50,043 unique publications remained. After a thorough screening of titles, abstracts, and, when necessary, full texts, the 200 most highly cited publications relevant to the topic were selected for analysis.

### 2.2 Inclusion and exclusion criteria

Inclusion Criteria: The title, abstract, and/or keywords clearly indicated direct relevance to migraine research. Peer-reviewed original research articles and review articles. Publications written in English. Publications with complete and extractable bibliographic metadata, including title, authors, institutional affiliations, publication year, journal name, and citation counts.

Exclusion Criteria: Publications identified as duplicates between WOS and PubMed databases (matched by DOI and title-author combination) were counted only once. Any publication that had been formally retracted at the time of data collection.

Duplicate records between WOS and PubMed were identified and removed using EndNote X9 through automated DOI matching, supplemented by manual verification of title-author combinations. After removing 48,260 duplicates, 50,043 unique publications remained.

The search results were sorted in descending order by citation count. Two independent researchers (GJ and XH) screened the titles and abstracts—and, when necessary, the full texts—to assess whether each publication met the inclusion criteria. Discrepancies between the researchers were resolved through consultation with a third researcher (SW.). Ultimately, the 200 most highly cited articles were selected for inclusion in the analysis.

The selection of the top 200 most highly cited publications was a deliberate methodological choice designed to balance comprehensiveness and focus. A larger sample size (e.g., top-500) would have included publications with substantially lower citation counts, potentially diluting the focus on the most influential and foundational works that define the field’s knowledge base and introducing excessive noise into network analyses. Conversely, a smaller sample (e.g., top-50 or top-100) might have been too restrictive, potentially missing seminal papers that have consistently demonstrated high impact across multiple research domains within migraine research (7). The top-200 threshold ensures a comprehensive overview of the core literature while maintaining a manageable dataset for in-depth bibliometric and network analyses using tools such as VOSviewer, CiteSpace, and bibliometrix (8).

### 2.3 Data analysis and visualization

This study employed multiple metrics—including the number of publications (NP), number of citations (NC), G-index, M-index, and total link strength (TLS)—to quantitatively assess the analysis targets from multiple dimensions. TLS quantifies the intensity of collaborative relationships between a given item and others (9). The G-index, a widely adopted bibliometric indicator, improves upon the conventional H-index by better capturing the impact of highly cited entities, making it more suitable for evaluating high-impact items in this study (10). Compared to the H-index, the M-index incorporates a temporal dimension, facilitating a more comprehensive assessment of academic contributions (11). Co-occurrence analysis, a common method for examining the simultaneous appearance of specific units within the literature, reveals relationships among them (12). Cluster analysis groups similar research objects based on shared characteristics (13). Co-citation refers to the occurrence of two or more documents being cited together by subsequent publications (14). Burstiness denotes the sudden emergence or marked increase in the frequency of specific items within a set time window (15). Historiographic analysis is employed to identify landmark publications within the field (16).

This study utilized the R package “bibliometrix” (4.3.2) (17) to visualize annual publication trends and conduct historiographic analysis, and assist in data curation. VOSviewer (1.6.19) (18) and Scimago Graphica (1.0.35) (19) were utilized for cluster analysis of relevant entities, and visualization of metrics including NP, NC, and TLS. CiteSpace (6.3. R3) (20) was applied to analyze references and keywords, as well as for constructing dual-map overlays of journals. Microsoft Excel 2021 was utilized for data organization and statistical calculations. Furthermore, no Artificial Intelligence–Generated Content tools were used to create, modify, or manipulate the original research data or results in this study.

## 3 Results

### 3.1 Literature analysis

**Figure 1:**
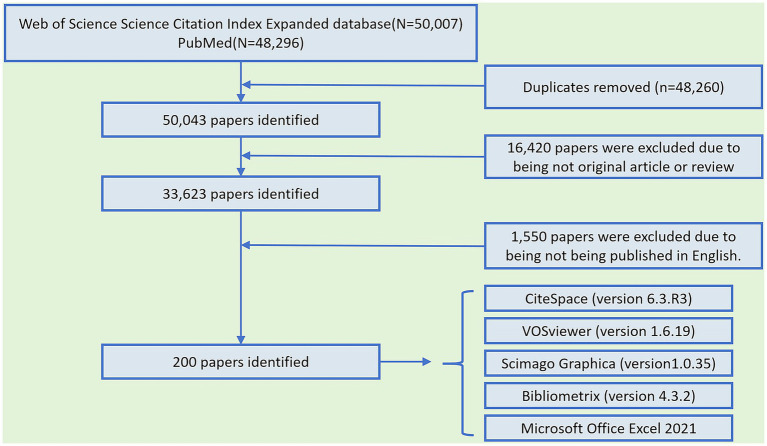
Search strategy.

A total of 50,007 publications were identified from WOS and 48,296 from PubMed in accordance with the search strategy outlined in Figure 1. After removing 48,260 duplicate records identified through DOI and title-author matching, 50,043 unique publications remained. After excluding non-article document types, 33,623 research articles and reviews remained. Restricting the results to English-language publications yielded a final dataset of 32,073 records. After sorting the records in descending order by cited count, manual screening was performed to identify the 200 most highly cited publications meeting the inclusion criteria. The most highly cited article was titled ‘Migraine Pathophysiology and Its Clinical Implications’, with a total of 8,699 citations. It provided a comprehensive overview of migraine pathophysiology, offered in-depth insights into its clinical relevance, and elucidated the mechanisms and therapeutic potential of pharmacological interventions (21). The second and third ranked publications were both issued by the International Headache Society, titled ‘Headache Classification Committee of the International Headache Society (IHS). The International Classification of Headache Disorders, 3rd edition’ and ‘The International Classification of Headache Disorders, 3rd edition (beta version),’ which received 5,453 and 3,415 citations, respectively. These two documents made significant contributions to the classification and diagnosis of headache disorders at different stages and have garnered widespread attention (22, 23). Each of the top three articles has been cited over 3,000 times, with a combined total of nearly 18,000 citations.

### 3.2 Analysis of publication trends

The observed peaks in NC and NP reflect distinct patterns of scientific impact. The NC peak in 2004 was primarily driven by the publication of ‘Migraine Pathophysiology and Its Clinical Implications’ (8,699 citations), which established a foundational framework for understanding migraine mechanisms. Similarly, the 2018 NC elevation corresponded to ‘Headache Classification Committee of the International Headache Society (IHS). The International Classification of Headache Disorders, 3rd edition’ (5,453 citations), which provided standardized diagnostic criteria widely adopted in clinical practice and research.

In contrast, the NP peak in 2010 represents a multidisciplinary convergence of breakthrough discoveries. This year witnessed simultaneous major advances across multiple research domains: the PREEMPT trials establishing OnabotulinumtoxinA for chronic migraine treatment (24), studies elucidating CGRP’s pathophysiological role (25) and photophobia mechanisms (26), genome-wide association studies identifying novel susceptibility loci (27), and the discovery of TRESK potassium channel mutations in familial migraine with aura (28). This convergence of foundational progress across multiple research domains resulted in an exceptionally high number of publications entering the top 200 list.

**Figure 2:**
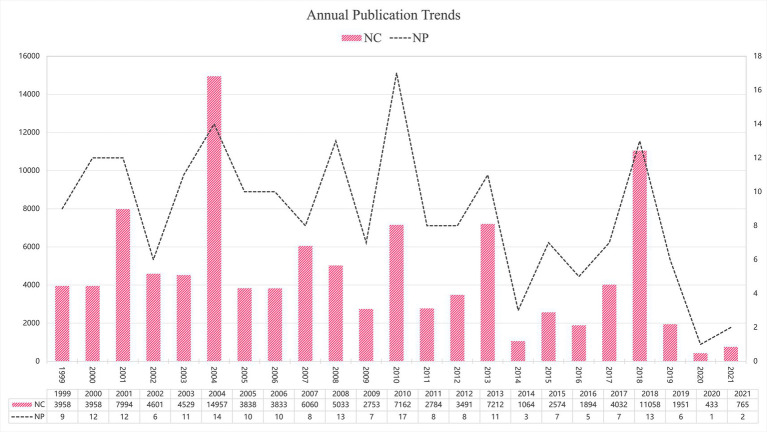
Annual publication trends.

Figure 2 illustrates the annual trends in NP and NC among the included studies. The highest NP was observed in 2010 (*N* = 17), while the peak NC occurred in 2004 (*N* = 14,957). In total, the 200 articles received 107,964 citations, averaging 539.82 citations per article, indicating substantial scholarly recognition within the field.

### 3.3 Journal analysis

**Figure 3:**
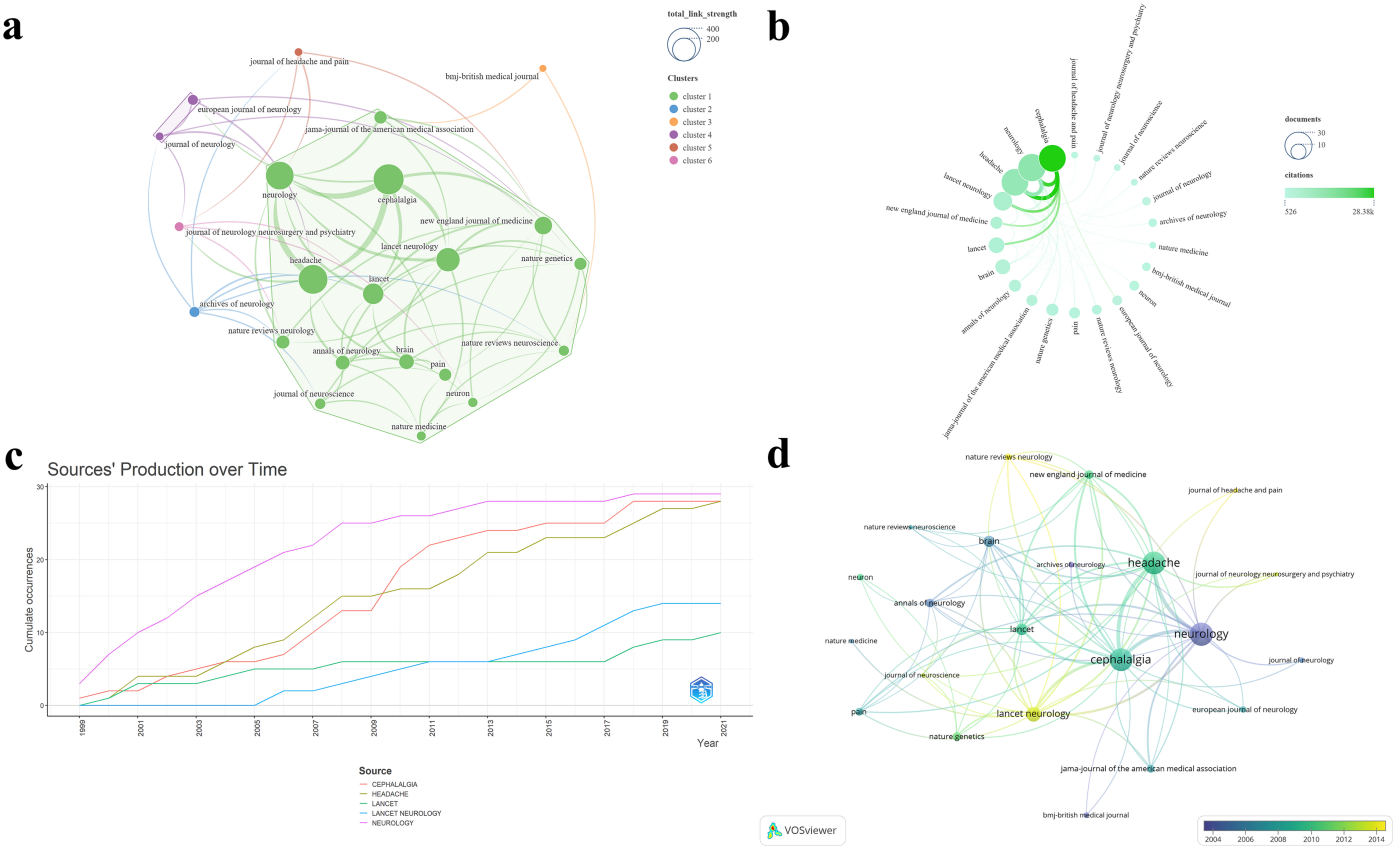
Visualization map of journals. **(a)** TLS and clustering; **(b)** NP and NC; **(c)** Annual publication trends of the top 5 journals by NP; **(d)** Temporal trends in journal publication volume.

A total of 45 journals were represented in this study. Among them, 22 journals with two or more publications were selected to construct a visualized map (Figure 3). The journals included in the analysis were primarily grouped into six clusters (Figure 3a). Table 1 lists the top five journals ranked by NP. The Journal Impact Factor (JIF) and JIF quartile data presented in the table are sourced from the 2023 Clarivate Journal Citation Reports.^3^ Figure 3b clearly illustrates the prominent advantages of Neurology, Cephalalgia, and Headache in both NP and NC. The journal with the highest NP was Neurology (*N* = 29), which also achieved the highest G-index (*N* = 29) and ranked within the top three across all evaluated metrics. Tied for second in NP were Cephalalgia (*N* = 28), representing the IHS, and Headache (*N* = 28), representing the American Headache Society. Among these, Cephalalgia exhibited the highest NC (*N* = 28,381), TLS (*N* = 323), and average citations per publication (*N* = 1,013.6). Conversely, HEADACHE attained the highest M-index (*N* = 1.077) and ranked second in both TLS (*N* = 297) and G-index (*N* = 28). Notably, all five journals are classified in Q1, with Lancet boasting the highest impact factor of 98.4. Figure 3c depicts the annual publication trends across journals, with Neurology, Cephalalgia, and Headache clearly maintaining dominant positions. The temporal evolution of journal publications reveals a positive growth trend for journals such as Lancet Neurology and Journal of Headache and Pain (Figure 3d). These journals have played a pivotal role in advancing knowledge accumulation and fostering innovation within the field.

**Table 1:** Journals with the highest NP.

Rank	Journals	NP	NC	Total Link strength	G-index	M-index	Average citations	JIF QUARTILE	JIF
1	Neurology	29	12,950	274	29	1.074	446.6	Q1	8.4
2	Cephalalgia	28	28,381	323	28	1.037	1013.6	Q1	5.0
3	Headache	28	11,075	297	28	1.077	395.5	Q1	5.3
4	Lancet Neurology	14	5,643	198	14	0.7	403.1	Q1	46.6
5	Lancet	10	4,132	154	10	0.385	413.2	Q1	98.4

**Figure 4:**
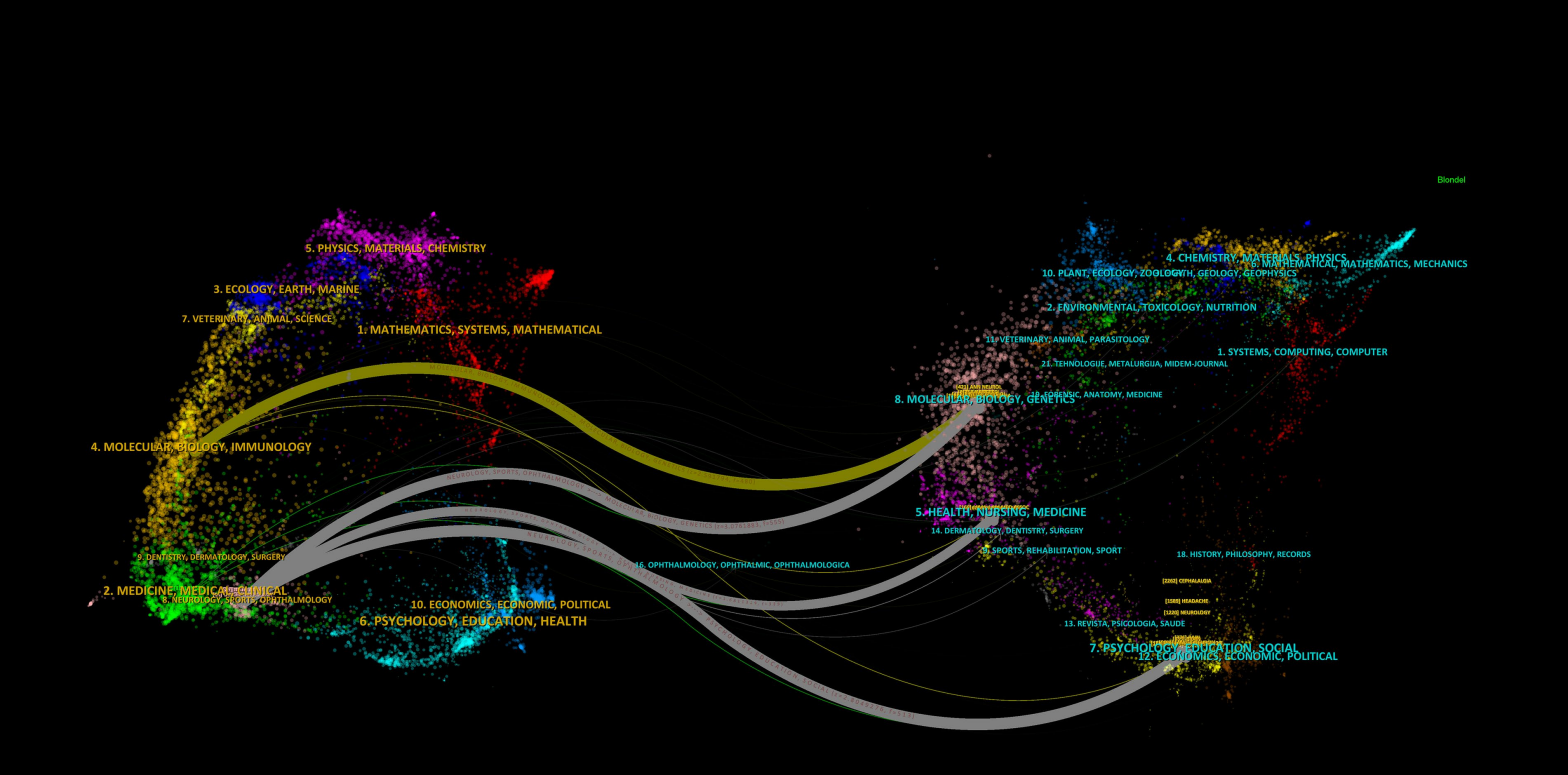
Dual-map overlay of journals. The citing journal map is shown on the left, and the cited journal map on the right. The connecting lines between them represent citation relationships between journals, with line thickness indicating the strength of citation linkage.

In the dual-map overlay of journals, the citing publications mainly stem from the domains of “Neurology/Sports/Ophthalmology” and “Molecular/Biology/Immunology.” These works cite studies within “Health/Nursing/Medicine,” “Molecular/Biology/Genetics,” and “Psychology/Education/Sociology” through four major citation pathways (Figure 4).

### 3.4 Author analysis

**Figure 5:**
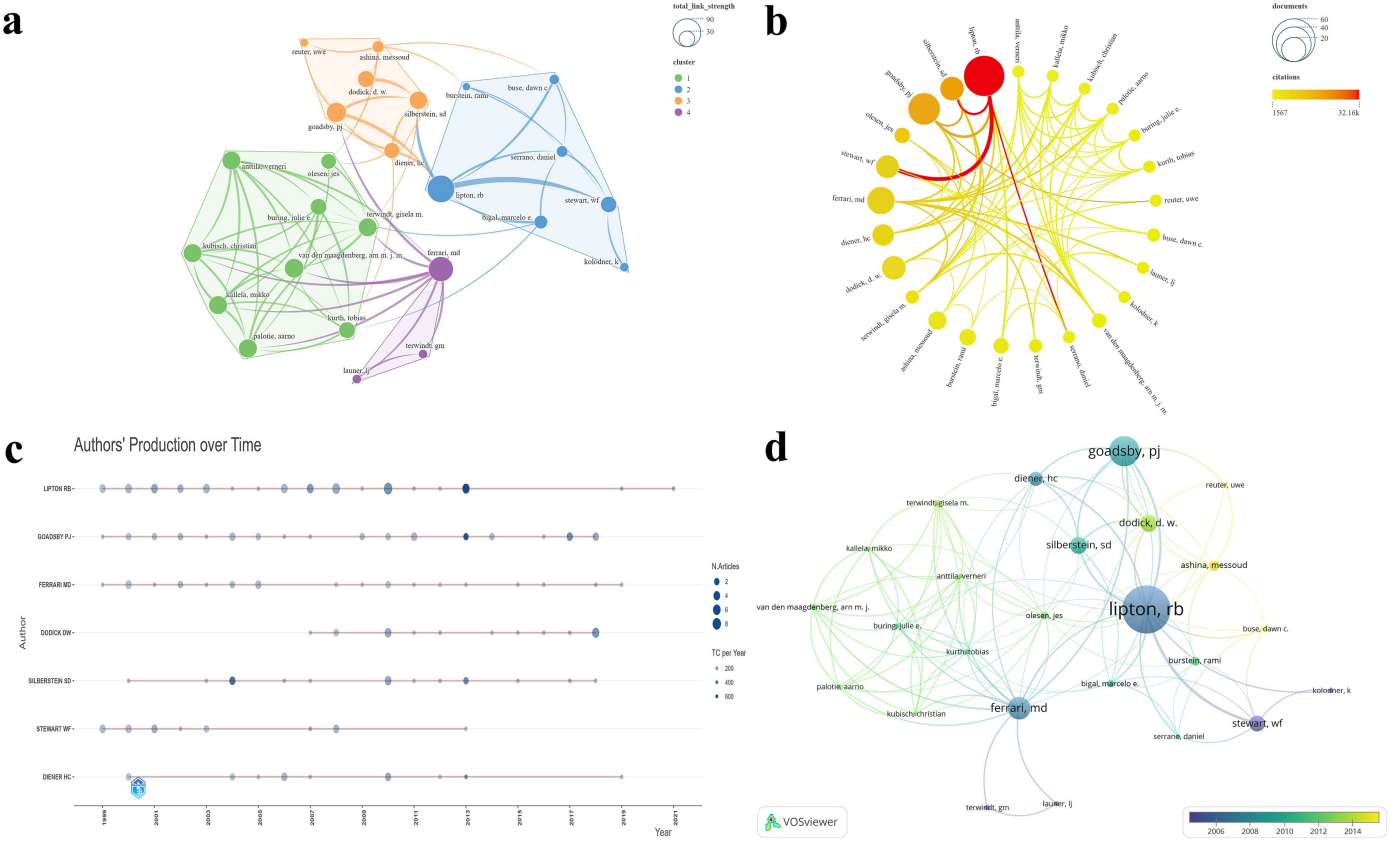
Visualization map of authors. **(a)** TLS and clustering; **(b)** NP and NC; **(c)** Annual publication trends of the top 7 authors by NP; **(d)** Temporal trends in author publication volume.

A total of 1,059 authors were involved in this study. Of these, 25 authors with five or more publications were selected for the construction of a visualized collaboration map (Figure 5). The authors included in the analysis were primarily grouped into four distinct clusters (Figure 5a). Table 2 presents the top five authors ranked by the NP. Figure 5b clearly highlights the prominent advantage of LIPTON RB, who ranked first across multiple metrics, including NP (*N* = 52), NC (*N* = 32,159), TLS (*N* = 80), G-index (*N* = 52), and M-index (*N* = 2). Additionally, LIPTON RB ranked third in average citations (*N* = 618.4). GOADSBY PJ ranked second in NP (*N* = 33), NC (N = 21,354), average citations (*N* = 647.1), G-index (*N* = 33), and M-index (*N* = 1.269). SILBERSTEIN SD achieved the highest average citations (*N* = 1080.8). The annual publication trends further demonstrate LIPTON RB’s sustained and significant influence within the field (Figure 5c). Moreover, the temporal evolution of author publication activity indicates that ASHINA, Messoud and BUSE, Dawn C. have emerged as some of the most active contributors in recent years (Figure 5d).

**Table 2:** Authors with the highest NP.

Rank	Authors	NP	NC	Total link strength	G-index	M-index	Average citations
1	Lipton RB	52	32,159	80	52	2	618.4
2	Goadsby PJ	33	21,354	42	33	1.269	647.1
3	Ferrari MD	24	10,013	66	24	0.923	417.2
4	Silberstein SD	18	19,455	35	18	0.72	1080.8
5	Dodick DW	18	7,361	30	18	1	408.9

### 3.5 Institutional analysis

**Figure 6:**
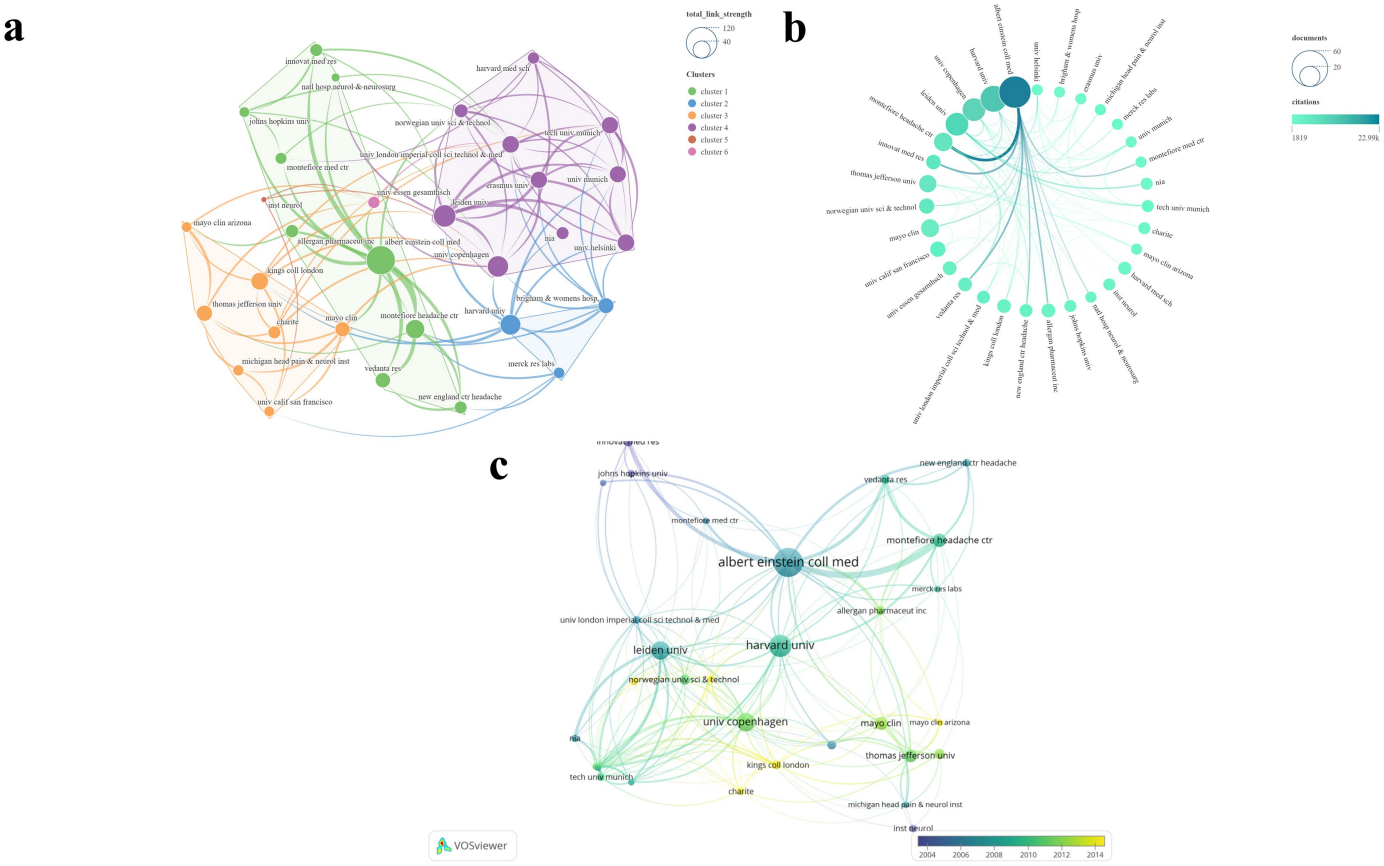
Visualization map of institutions. **(a)** TLS and clustering; **(b)** NP and NC; **(c)** Temporal trends in institution publication volume.

A total of 453 institutions were involved in this study. Of these, 31 institutions with six or more publications were selected for the construction of a visualized mapping (Figure 6). The institutions included in the analysis were primarily grouped into six clusters (Figure 6a). Table 3 presents the top five institutions ranked by NP. Figure 6b clearly illustrates the prominent advantage of Albert Einstein Coll Med, which ranked first with the highest scores in NP (*N* = 45), NC (N = 22,987), and average citations (N = 510.8). Harvard Univ ranked second in both NP (*N* = 31) and NC (N = 12,816). In terms of TLS, Leiden Univ ranked first (*N* = 243), followed closely by the Univ Copenhagen (*N* = 236). Additionally, Montefiore Headache Ctr achieved the second highest average citations (*N* = 433.9). The temporal evolution of institutional publications highlights Kings Coll London and Mayo Clin Arizona as leading institutions at the forefront of the field (Figure 6c).

**Table 3:** Institutions with the highest NP.

Rank	Institutions	NP	NC	Total link strength	Average citations
1	Albert Einstein Coll Med	45	22,987	213	510.8
2	Harvard Univ	31	12,816	178	413.4
3	Univ Copenhagen	24	10,274	236	428.1
4	Leiden Univ	24	9,789	243	407.9
5	Montefiore Headache Ctr	16	6,943	72	433.9

### 3.6 Countries/regions analysis

**Figure 7:**
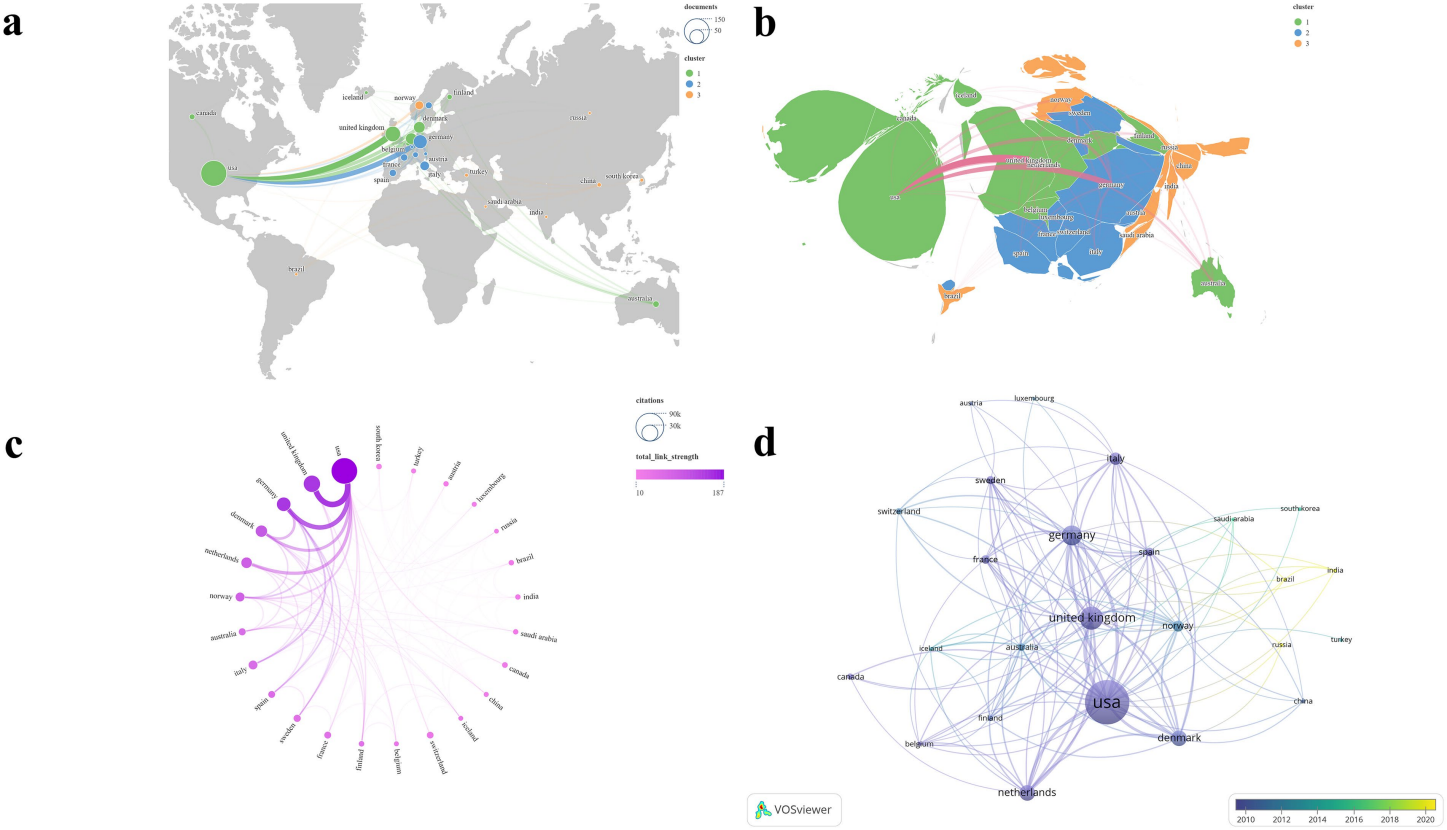
Visualization map of countries/regions. **(a,b)** NP and clustering; **(c)** TLS and NC; **(d)** Temporal trends in countries/region publication volume.

After standardizing the names of countries and regions, we selected 25 countries/regions with two or more publications out of the total 45 for the visualization mapping (Figure 7). The countries/regions included in the analysis were primarily grouped into seven clusters (Figures 7a,b). Table 4 presents the top six countries/regions ranked by NP. Figure 7c clearly illustrates the United States’ dominant lead in both NP and NC. The U. S. has established the most extensive national collaboration network, centered around Europe and North America. The United States ranked first, contributing 148 publications—nearly equal to the combined total of the next four countries/regions. In addition, the United States recorded the highest NC (*N* = 73,089) and TLS (*N* = 187), while also securing the second-highest average citation (*N* = 493.8). The United Kingdom achieved the highest average citation (*N* = 562.1) and ranked second in terms of NP (*N* = 52), NC (*N* = 29,231), and TLS (*N* = 160). The temporal evolution of publications by country/region reveals that India and Brazil have become increasingly active in this field in recent years (Figure 7d).

**Table 4:** Countries/Regions with the highest NP.

Rank	Countries	NP	NC	Total link strength	Average citations
1	United States	148	73,089	187	493.8
2	United Kingdom	52	29,231	160	562.1
3	Germany	42	19,661	158	468.1
4	Denmark	29	13,508	109	465.8
5	Netherlands	29	11,390	98	392.8

### 3.7 Research categories analysis

The included publications were categorized into various research directions according to the WOS categories. The most represented categories were Clinical Neurology (*N* = 142) and Neurosciences (*N* = 68).

### 3.8 Keyword analysis

**Figure 8:**
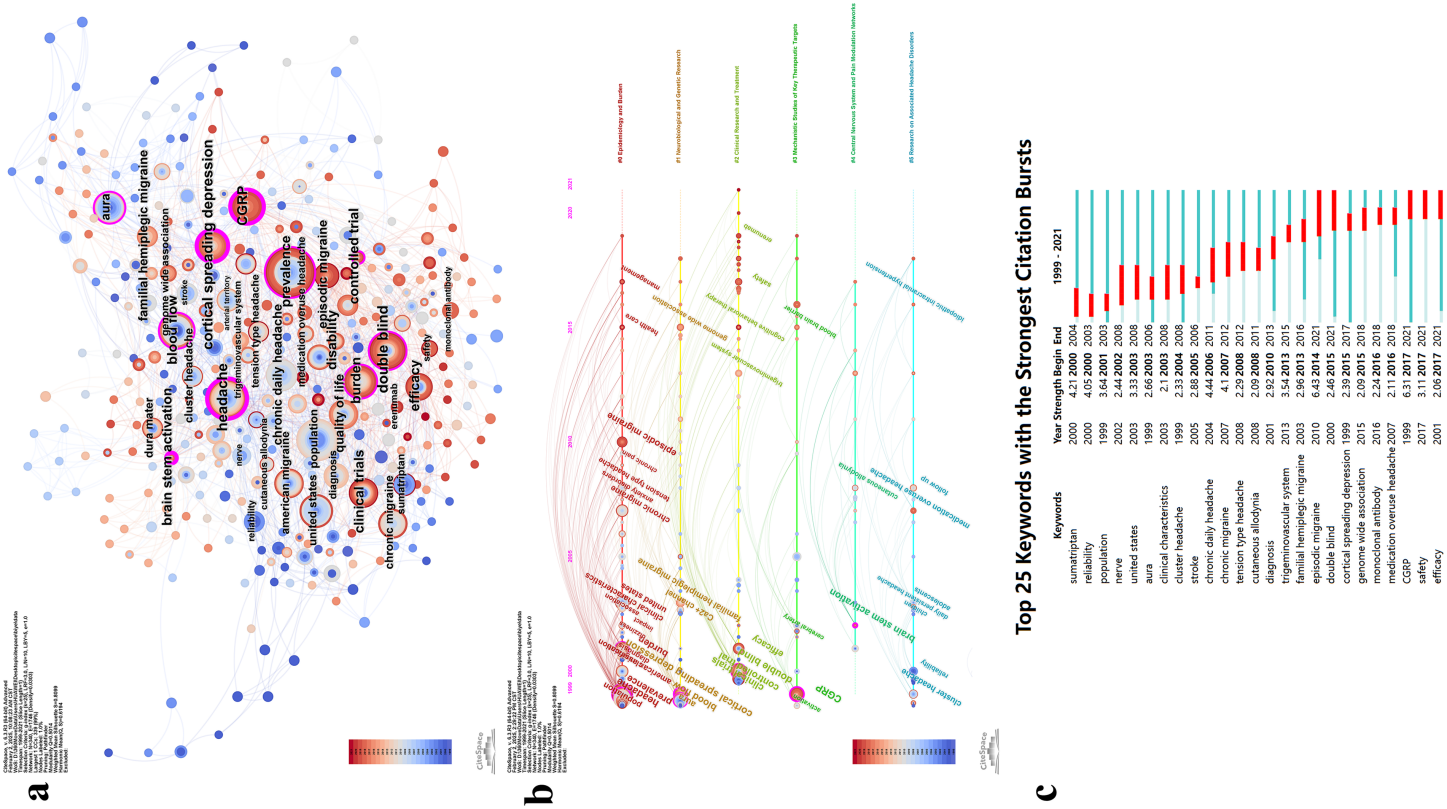
Visualization map of keywords. **(a)** Keyword co-occurrence map; **(b)** Keyword timeline view; **(c)** Infographic of top burst keywords.

After merging synonymous keywords, a total of 860 unique terms were identified. Using CiteSpace with a scale factor of K = 25, 340 keywords were selected for the final analysis (Figure 8, Table 5). The keyword co-occurrence map is presented in Figure 8a. The most frequently occurring keywords included prevalence (*N* = 54), headache (*N* = 46), double blind (*N* = 36), CGRP (*N* = 35), population (*N* = 34), burden (*N* = 32), cortical spreading depression (*N* = 31), quality of life (*N* = 25), disability (*N* = 22), episodic migraine (*N* = 21), familial hemiplegic migraine (*N* = 20), brain stem activation (*N* = 19), controlled trial (*N* = 17), pain (*N* = 17), blood flow (*N* = 16), united states (*N* = 16), chronic daily headache (*N* = 15), clinical trials (*N* = 14), aura (*N* = 14), chronic migraine (*N* = 14), efficacy (*N* = 13), epidemiology (*N* = 12), diagnosis (*N* = 12), clinical characteristics (*N* = 11), and sumatriptan (*N* = 11). Keyword clustering analysis identified six major clusters. The cluster labels were manually refined based on the thematic content of the included keywords. The largest cluster was Cluster #0: Epidemiology and Burden, followed by Cluster #1: Neurobiological and Genetic Research, Cluster #2: Clinical Research and Treatment, Cluster #3: Mechanistic Studies of Key Therapeutic Targets, Cluster #4: Central Nervous System and Pain Modulation Networks, and Cluster #5: Research on Associated Headache Disorders.

**Table 5:** Major keywords and cluster.

Rank	Keywords	Count	Cluster ID	Mean (Year)	Representative content
1	Prevalence	54	0	2005	Prevalence (53), headache (45), population (34), burden (32), quality of life (25), disability (22)
2	Headache	46	1	2005	Cortical spreading depression (31), familial hemiplegic migraine (20), blood flow (16), aura (14), Ca2 + channel (8), mechanisms (5), episodic vertigo (4), genome wide association (4)
3	Double Blind	36	2	2010	Double blind (36), controlled trial (17), clinical trials (14), efficacy (13), sumatriptan (11), safety (6), trigeminovascular system (6), cognitive behavioral therapy (3), erenumab (3), acute oral treatment (3)
4	CGRP	35	3	2009	CGRP (35), stroke (7), activation (6), neurogenic inflammation (4), monoclonal antibody (4), blood brain barrier (3), cerebral artery (3), extracerebral circulation (3)
5	Population	34	4	2010	Brain stem activation (19), cutaneous allodynia (6), cortex (4), activity modifying protein 1(3), dorsal horn (2), hypothalamic activation (2)
6	Burden	32	5	2005	Cluster headache (10), reliability (8), medication overuse headache (5), adolescents (4), follow up (4), daily persistent headache (3), chronic paroxysmal hemicrania (3), comorbidity (3)

An analysis of keyword trends over time revealed distinct thematic shifts across four periods (Figure 8b). Between approximately 1999 and 2005, research initially engaged with all six major clusters, establishing foundational knowledge in epidemiology (prevalence, burden, disability), pathophysiology (CGRP, cortical spreading depression, brain stem activation), and evidence-based treatment evaluation (double blind, controlled trials, efficacy).

From around 2005 to 2010, research broadened to encompass migraine subtypes (episodic migraine, chronic migraine, tension-type headache) and comorbidities (anxiety disorders, cardiovascular disease, MOH). Advanced neuroimaging techniques (voxel-based morphometry) and emerging therapeutic targets (cyclase-activating polypeptide, topiramate) gained prominence, alongside attention to special populations (school children) and clinical phenomena (cutaneous allodynia).

Between approximately 2010 and 2015, the field demonstrated maturation through genetic investigations (genome wide association, susceptibility loci), mechanistic insights (trigeminovascular system, blood–brain barrier, activity modifying protein 1), and therapeutic innovations (cognitive behavioral therapy, preventive treatment strategies). Healthcare delivery and assessment tools (MIDAS questionnaire) also received increased attention.

After 2016, research focus narrowed primarily to Cluster #2 (Clinical Research and Treatment) and Cluster #3 (Mechanistic Studies of Key Therapeutic Targets). The CGRP system dominated this period (monoclonal antibody, CGRP receptor antagonists, tolerability), supported by translational research (mouse models, gene expression, functional connectivity) and expanded clinical trial methodologies (community-based studies, healthy subjects). This shift toward precision medicine marked a transition from broad mechanistic exploration to focused therapeutic development.

Analysis of the 25 keywords with the strongest burstiness revealed that episodic migraine exhibited the highest burst strength (*N* = 6.43), followed by CGRP (*N* = 6.31) and chronic daily headache (*N* = 4.44). Keywords with burstiness persisting to the present include episodic migraine (*N* = 6.43), double blind (*N* = 2.46), CGRP (*N* = 6.31), safety (*N* = 3.11), and efficacy (*N* = 2.06) (Figure 8c).

### 3.9 Reference analysis

**Figure 9:**
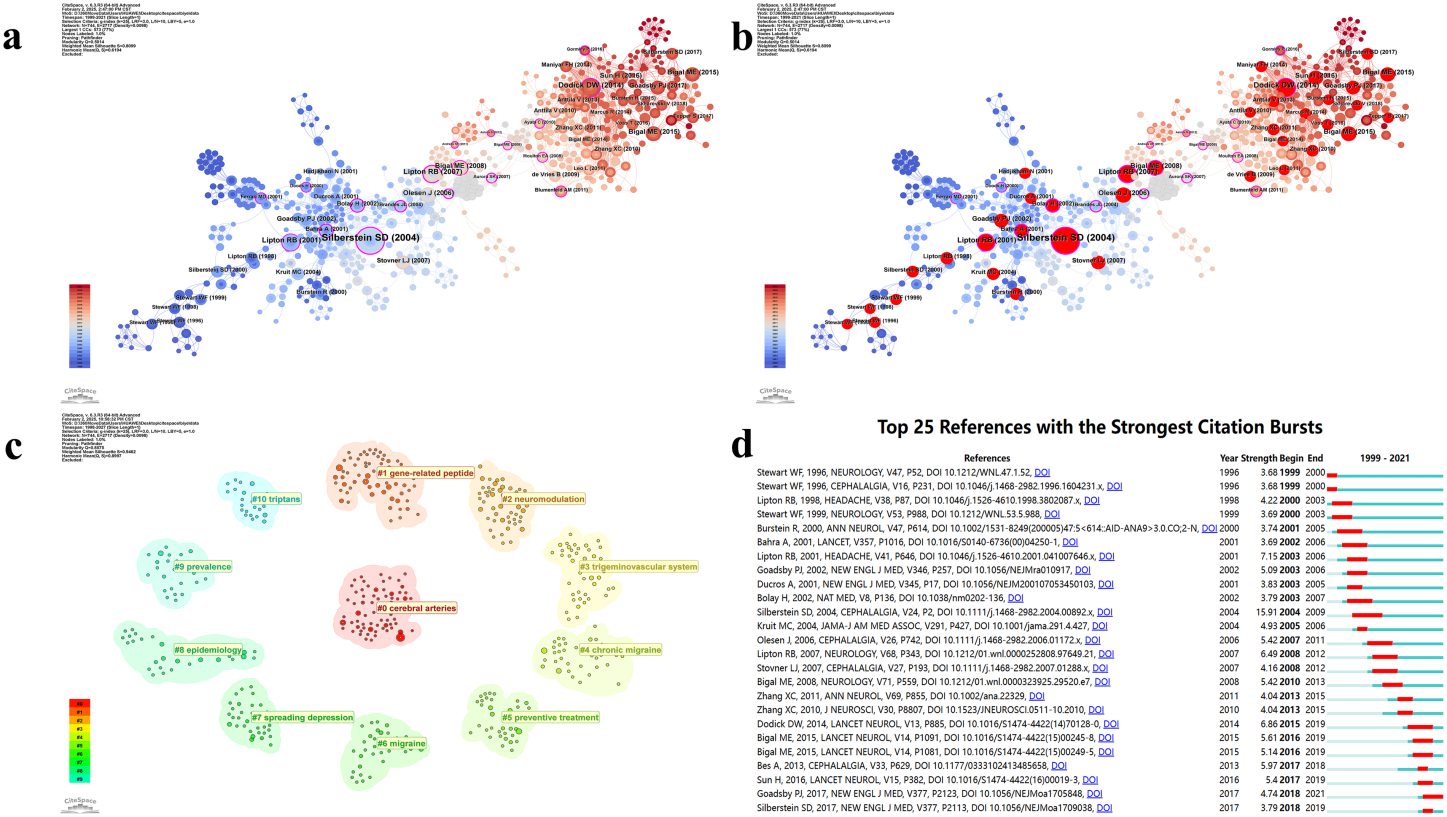
Visualization map of references. **(a)** Reference co-occurrence map; **(b)** Reference co-occurrence map with bursty references highlighted; **(c)** Reference cluster map; **(d)** Infographic of top burst references.

Using a scale factor of K = 25 in CiteSpace, 744 references were selected from a total of 7,995 citations for inclusion in the analysis (Figure 9, Table 6). The co-occurrence network of references is presented in Figures 9a,b. The most cited reference is “Migraine pathophysiology and its clinical implications,” with 37 citations, followed by “Safety and efficacy of LY2951742, a monoclonal antibody to calcitonin gene-related peptide, for the prevention of migraine: a phase 2, randomized, double-blind, placebo-controlled study,” which has 16 citations. Using Latent Semantic Indexing, labels for 11 major clusters were identified. The largest cluster was Cluster #0: cerebral arteries, followed by Cluster #1: gene-related peptide, Cluster #2: neuromodulation, Cluster #3: trigeminovascular system, Cluster #4: chronic migraine, Cluster #5: preventive treatment, Cluster #6: migraine, Cluster #7: spreading depression, Cluster #8: epidemiology, Cluster #9: prevalence, and Cluster #10: triptans (Figure 9c). Subsequently, burstiness analysis was conducted, with nodes highlighted by red circles indicating terms exhibiting burstiness (Figures 9b,d).

**Table 6:** Major references and clusters.

Rank	Cited reference	Count	Journals	Cluster ID	Mean (Year)	Label (LSI)
1	Migraine pathophysiology and its clinical implications	7	Cephalalgia	0	2001	Cerebral arteries; h alpha cgrp; botulinum toxin type; consensus statement; periaqueductal gray | botulinum toxin type; trigeminal nerve; cerebral arteries; h alpha cgrp; consensus statement
2	Safety and efficacy of LY2951742, a monoclonal antibody to calcitonin gene-related peptide, for the prevention of migraine: a phase 2, randomized, double-blind, placebo-controlled study	16	Lancet Neurol	1	2016	Gene-related peptide; trigeminal system; nitric oxide; primary afferent neurons; acute | acute; treatment; migraine; preventive; principles
3	Prevalence and burden of migraine in the United States: data from the American Migraine Study II	14	Headache	2	2012	Neuromodulation; trigeminovasculature; neuropeptide; sensitization; photophobia | trigeminovascular pathway; premonitory phase; cortical spreading depression; neuromodulation; trigeminovasculature
4	Migraine prevalence, disease burden, and the need for preventive therapy	14	Neurology	3	2009	Trigeminovascular system; cortical spreading depression; meningeal nociceptors; neuronal excitability; pain modulation | inhibitory balance; neurogenic inflammation; spreading depression; cortical spreading depression; meningeal nociceptors
5	Safety, tolerability, and efficacy of TEV-48125 for preventive treatment of chronic migraine: a multicentre, randomized, double-blind, placebo-controlled, phase 2b study	12	Lancet Neurol	4	2010	Chronic migraine; international survey; episodic migraine; psychiatric disorders | psychiatric disorders; episodic migraine; international survey; chronic migraine
6	New appendix criteria open for a broader concept of chronic migraine	12	Cephalalgia	5	2004	Preventive treatment; chronic daily headache; clinical trial; randomized trial; chronic migraine treatment | randomized trial; chronic migraine treatment; clinical trial; preventive treatment; chronic daily headache
7	The International Classification of Headache Disorders, 3rd edition (beta version)	11	Cephalalgia	6	2004	Migraine; brainstem; orexin; trigeminal; dopamine | modifiable risk factors; migraine progression; magnetic resonance imaging; migraine; brainstem
8	Chronic migraine in the population: burden, diagnosis, and satisfaction with treatment	11	Neurology	7	2012	Spreading depression; trigeminovascular system; neurogenic inflammation; excitatory/inhibitory balance
9	Safety and efficacy of AMG 334 for prevention of episodic migraine: a randomized, double-blind, placebo-controlled, phase 2 trial	11	Lancet Neurol	8	1996	Epidemiology; disability; headache; migraine; American migraine study | American migraine study; epidemiology; migraine; disability; migraine disability
10	Safety, tolerability, and efficacy of TEV-48125 for preventive treatment of high-frequency episodic migraine: a multicentre, randomized, double-blind, placebo-controlled, phase 2b study	11	Lancet Neurol	9	2005	Prevalence; treatment; migraine; prevention; mental health disorders | septal defects; migraine disorders; prevalence; treatment; migraine
11	Migraine--current understanding and treatment	10	New Engl J Med	10	1998	Triptans; migraine; serotonin agonists; treatment; metaanalysis; clinical trials

### 3.10 Landmark literature analysis

**Figure 10:**
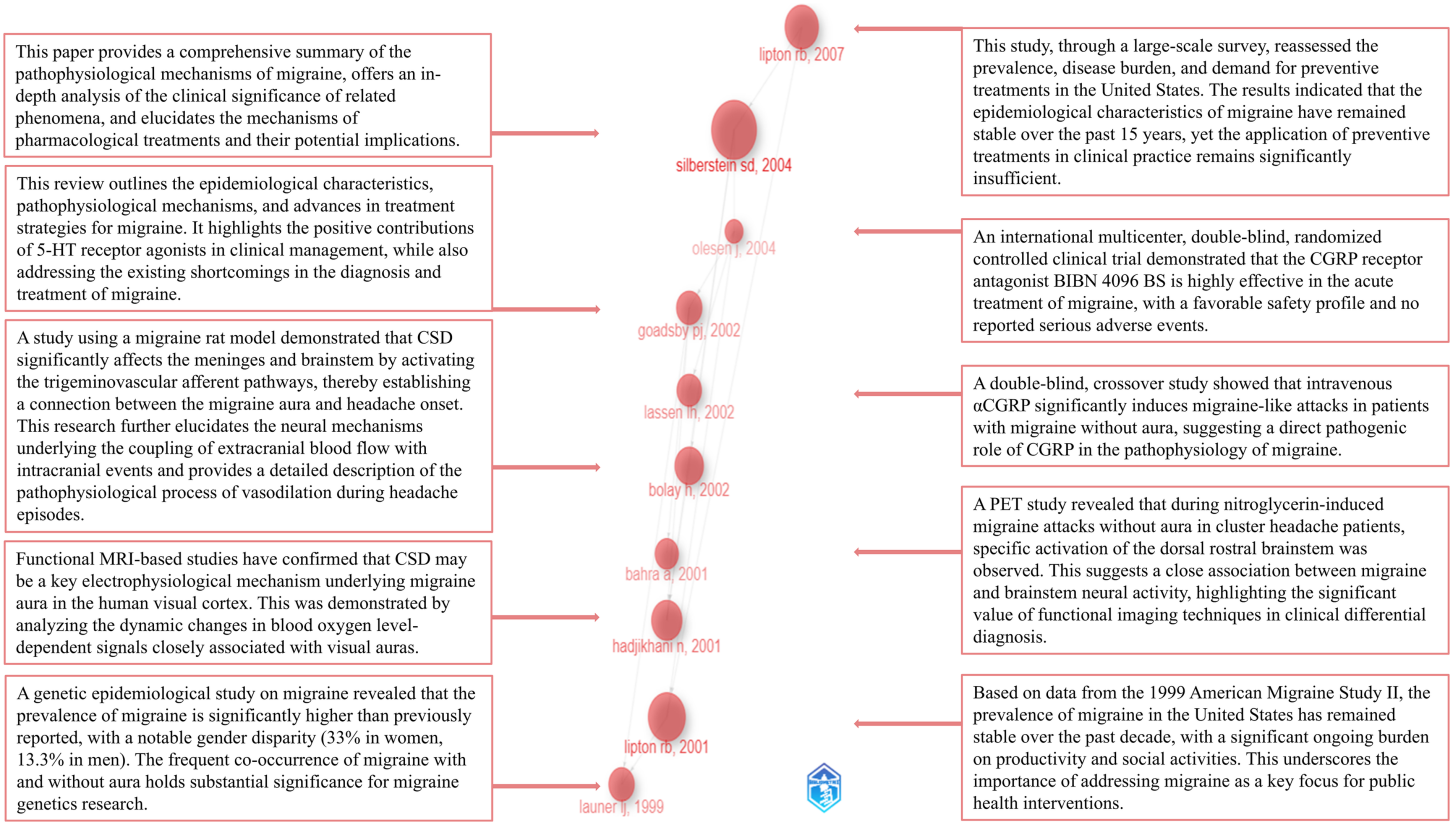
Landmark publications and their major contributions.

The R package bibliometrix was employed to identify the 10 most influential landmark publications from the included dataset. Figure 10 presents an overview of these publications, including their year of publication, leading authors, and a brief summary of their research focus.

## 4 Discussion

### 4.1 General information

This study is the first to employ bibliometric analysis to comprehensively examine the development and emerging trends of the 200 most influential publications on migraine indexed in both the SCI-E and PubMed databases between January 1, 1999, and December 7, 2024. The analysis encompassed 45 journals, 1,059 authors, 45 countries/regions, 453 institutions, 860 keywords, and 7,995 cited references.

In the journal analysis, Neurology, Cephalalgia, and Headache emerged as the three most prominent journals in the domain of migraine research across all evaluated metrics. These journals demonstrate clear leadership in TLS and NC, reflecting their extensive collaborative networks and substantial academic impact. Notably, Cephalalgia exhibited the highest NC and average citations, underscoring its rigorous publication standards and broad recognition among experts. HEADACHE achieved the highest M-index, reflecting not only the high quality of its publications but also their sustained impact over time. Emerging journals such as Lancet Neurology and Journal of Headache and Pain are progressively gaining prominence. All of the top five journals by NP are classified as Q1 journals, further emphasizing the exceptional level of evidence within this domain (Figure 3; Table 1). Figure 4 illustrates the complex relationships between citing and cited references, highlighting the multidisciplinary and interconnected nature of the research. This complexity underscores the necessity for researchers to adopt a broad and integrative perspective across diverse topics and fields.

Author analysis revealed that LIPTON RB consistently leads across all evaluated metrics, demonstrating sustained and significant contributions, and is recognized as one of the most influential authors in the field. GOADSBY PJ consistently ranks second on multiple key indicators, similarly making substantial contributions to the discipline’s advancement. Notably, SILBERSTEIN SD holds the highest average citation and ranks third in NC, reflecting widespread recognition of his high-quality work. Emerging authors such as ASHINA, MESSOUD, and BUSE, DAWN C. are progressively gaining prominence (Figure 5; Table 3).

In the institutional analysis, Albert Einstein Coll Med emerges as the most influential institution in the field, demonstrating a clear advantage across all evaluated metrics. Leiden Univ and Univ Copenhagen have established broad collaborative networks, securing widespread recognition in the field. However, Montefiore Headache Ctr would benefit from further expanding its network and fostering greater collaboration. Institutions such as King’s College London and Mayo Clinic Arizona stand out as prominent leaders at the forefront of the field, warranting sustained attention (Figure 6; Table 4).

The country/region analysis reveals that the majority of publications are concentrated in North America and Europe. Based on the various metrics included in the analysis, the United States holds a dominant position in the field, establishing itself as the most influential country in migraine research. The United Kingdom, with the highest average citation, ranks second in all other metrics, indicating that its publications have garnered significant recognition and made substantial contributions to the field. Countries such as India and Brazil have demonstrated a steady increase in influence in recent years, emerging as prominent nations at the forefront of the field and warranting continued attention (Figure 7; Table 3).

### 4.2 Knowledge Base

The analysis of landmark literature, in conjunction with the interpretation of keyword and reference analysis results, offers a more comprehensive understanding of the field’s evolution. This also facilitates well-founded predictions about its future trajectory.

An analysis of the most frequently occurring keywords reveals that researchers have focused on a range of migraine types and their associated symptoms. Scholars from diverse disciplines, including epidemiology, physiology, and pathology, have focused on critical areas such as disease diagnosis, treatment strategies, and patient health outcomes. Their efforts are directed toward generating robust, evidence-based insights into migraine treatment approaches through rigorous and methodologically sound research. Between approximately 1999 and 2005, researchers concentrated on the epidemiology, disease burden, and impact of migraine on patient quality of life. Emphasizing the importance of rigorous research methodologies, such as double-blind and randomized controlled trials, they underscored their vital role in enhancing the quality of evidence. The central role of accurate diagnosis and preventive treatments in migraine management was confirmed. Additionally, various therapies, including sumatriptan, botulinum toxin A, and other treatments, were thoroughly evaluated. These studies highlighted the significance of factors such as CGRP, cortical spreading depression (CSD), brainstem activation, blood flow, and the dura mater in the pathophysiology of migraine. Additionally, disease types such as migraine with aura, cluster headaches, and familial hemiplegic migraine, along with their clinical characteristics, have garnered significant attention. Between approximately 2005 and 2010, researchers focused on various types of headache/migraine, including episodic migraine, chronic migraine, and tension-type headaches, as well as associated conditions such as cardiovascular diseases and anxiety disorders. The significance of voxel detection and follow-up assessments has been firmly established. Researchers have focused on the significance of adenylyl cyclase-activating polypeptides, neurogenic plasma extravasation, hypothalamic activation, cervical spinal cord, and dorsal horn in the pathophysiology of migraine. The efficacy and safety of pharmacotherapies, including topiramate and ergot alkaloids, have been further evaluated. Additionally, special populations, such as children, have garnered significant interest from researchers. Additionally, symptoms such as abnormal cutaneous pain perception, cognitive dysfunction, and chronic pain, as well as severe drug side effects like medication overuse headache, have also received significant attention from researchers. Between approximately 2010 and 2015, genome-wide association studies (GWAS) gradually emerged as a powerful tool, making substantial contributions to the genetic research of migraine. Menstrual migraine gained increasing attention from researchers during this period. Early intervention for patients, along with the role of cognitive behavioral therapy, has garnered growing interest. The use of questionnaires in healthcare settings expanded further and continued to be refined. Factors such as the central nervous system, trigeminovascular system, blood–brain barrier (BBB), and Receptor Activity Modifying Protein 1, have been increasingly studied for their roles in the pathophysiological progression of migraine. Since 2016, the application of CGRP monoclonal antibodies and their receptor antagonists has garnered widespread attention from researchers, becoming a major focus of study. Clinical and animal studies on the safety, efficacy, and tolerability of these drugs have been continuously conducted. The recruitment of healthy controls and community-based research have received significant emphasis. Further exploration into the regulatory role of genes in migraine has been undertaken. Increased focus on C-reactive protein and the dorsal root ganglia has deepened researchers’ understanding of migraine pathophysiology. Additionally, idiopathic intracranial hypertension and the cyclical nature of migraine have sparked strong interest among researchers.

The Burstiness analysis of keywords indicates that episodic migraine will remain a central focus of research in the present and foreseeable future. Researchers will continue to delve into the mechanisms and drug development targeting the CGRP system, which serves as a key therapeutic target for migraine. Chronic daily headache, along with the safety and efficacy of various treatments, will remain central to clinical evaluation. Research methodologies, such as double-blind trials, will continue to serve as essential tools for assessing clinical efficacy (Figure 8; Table 5).

As shown in Figure 10, we identified the 10 landmark papers in the field through a historiographic analysis, summarizing their publication years and key findings (21, 29–37). This analysis offers valuable insights into the developmental trajectory of the field. By synthesizing the key findings from the 10 landmark papers, it is evident that researchers have focused on elucidating the pathophysiological mechanisms of migraine (21, 32, 35, 36). Key phenomena such as CSD, migraine with aura, vasodilation, and brainstem activation have received particular attention (29, 32, 33). Structures like the trigeminovascular system, visual cortex, brainstem, and medulla oblongata are considered to play pivotal roles in the progression of migraine (29, 32, 33). Positron emission tomography (PET) and functional MRI (fMRI) have seen increasing widespread application in research (29, 33). The pro-nociceptive role of CGRP has been firmly established (31), and drugs targeting the CGRP system have achieved significant success, becoming a new focal point of research (30). Additionally, researchers have consistently maintained close attention to disease burden and epidemiology (34, 37). A substantial number of high-quality clinical trials have been continuously conducted (30, 31). Notably, all 10 landmark papers in this field were published approximately a decade ago, highlighting that the foundational efforts of previous researchers have established a solid theoretical base for the field. In addition to the 10 landmark papers mentioned above, researchers have also focused on the differential diagnosis of migraine subtypes, including chronic migraine, episodic migraine, and familial hemiplegic migraine (22, 23, 38–40). The positive impact of preventive treatments for migraine has been firmly established (41). A variety of research methodologies, including systematic reviews, randomized controlled trials and meta-analyses, have been widely applied, contributing to the establishment of a robust evidence quality framework (39, 42). Clinical efficacy and safety of various therapies, such as triptans and botulinum toxin type A, have been evaluated extensively (24, 39). Notably, acupuncture, recognized as a safe and low-side-effect treatment, has garnered widespread attention (41, 43, 44). Furthermore, the efficacy and safety of several other CGRP monoclonal antibodies and receptor antagonists have been systematically evaluated and validated, providing a solid foundation for their further large-scale clinical application (45, 46).

Figure 9 and Table 6 presents the results of the reference analysis. The most cited papers in the field similarly highlight researchers’ close attention to migraine burden, epidemiology, and disability (36). Large-scale questionnaires have been employed, with continuous efforts to improve communication between healthcare providers and patients in order to obtain more precise data (47, 48). Disease diagnosis and preventive treatment have received considerable attention (23, 34, 40). At the same time, the emphasis on the pathophysiological mechanisms of the disease and related anatomical knowledge has enabled researchers to achieve satisfactory therapeutic outcomes grounded in a solid theoretical foundation (21, 49). A substantial number of clinical trials have been conducted to assess the safety, efficacy, and tolerability of various CGRP-targeting drugs across different migraine subtypes, including episodic and chronic migraine (50–52).

At the genetic level, technologies such as GWAS have been widely applied to identify gene function associations and susceptibility loci, providing deep insights into the genetic mechanisms of the disease and offering valuable clues for potential therapeutic targets (53). In addition, researchers’ focus has extended to subcellular structures, such as the Panx1 channels and calcium channels in neurons (54, 55). Furthermore, in-depth investigations have been conducted into phenomena such as disease-related brain effects and allodynia (56, 57). One reference continues to exhibit sustained high burstiness at present, with a study demonstrating, through a randomized, double-blind, placebo-controlled trial, that monthly subcutaneous injections of erenumab significantly reduce the frequency of migraine attacks, improve the impact of migraine on daily activities, and decrease the use of acute medications (46).

### 4.3 Research focus

The clustering analysis of references and keywords highlights the primary research focuses. In conjunction with the results of the clustering analysis and our understanding of the field, we have briefly summarized several key research hotspots: clinical treatments, clinical translation of key therapeutic targets, genetics of migraine, the trigeminovascular system and pain regulation networks in migraine, neuroimmunology, aura, and comorbidities.

#### 4.3.1 Clinical treatments

In clinical practice for migraine, pharmacological treatment remains the primary approach to managing the condition. Common pharmacological options for preventive treatment include antiepileptics, tricyclic antidepressants, and beta-blockers. In the acute phase, triptans and nonsteroidal anti-inflammatory drugs (NSAIDs) are typically considered first-line treatments. In severe cases, opioids may even be used in combination (58).

It is noteworthy that traditional herbal medicines have long encountered challenges in achieving widespread international recognition largely due to their reliance on relatively abstract theoretical frameworks. However, in recent years, the extensive application of modern technologies—such as liquid chromatography-mass spectrometry (LC–MS), metabolomics, network pharmacology, and molecular docking—has increasingly uncovered their physicochemical properties, providing a solid foundation for validating their clinical efficacy and promoting broader acceptance (59). In addition, non-pharmacological treatments have garnered increasing attention in recent years. Therapies such as acupuncture and transcranial electrical stimulation have gradually gained academic recognition through the conduct of numerous high-quality clinical studies (41, 60). Surgical approaches targeting peripheral nerve decompression and deactivation of trigger sites in adjacent tissues, such as muscles and fascia, have also garnered significant interest from researchers (61). However, the lack of high-quality evidence has limited the widespread application and development of these therapies. In the future, conducting large-scale, long-term, multi-center randomized controlled trials and real-world studies will provide more comprehensive and reliable evidence, facilitating a better evaluation of these therapies.

#### 4.3.2 Clinical translation of key therapeutic targets

The role of the 5-hydroxytryptamine (5-HT) system in migraine has long been well-established (62), particularly with the extensive clinical use and widespread recognition of several triptans targeting the 5-HT1B/1D receptors (58). Similarly, 5-HT₁F receptor agonists, exemplified by lasmiditan, have also achieved significant success (63). However, efforts to update and optimize medications are ongoing. Both CGRP and pituitary adenylate cyclase-activating polypeptide (PACAP) exhibit potent vasodilatory effects and can induce neurogenic inflammation through mechanisms such as mast cell degranulation and the release of inflammatory mediators (64, 65). Migraine patients consistently report elevated levels of CGRP and PACAP (66). Furthermore, extensive evidence demonstrates that exogenous infusion of both substances can trigger migraine-like headaches in patients (67, 68). Drugs targeting the CGRP system have undoubtedly become a focal point in contemporary migraine treatment. The development of CGRP-targeted therapies represents a paradigm shift in migraine management. The first CGRP monoclonal antibody, erenumab (Aimovig), received FDA approval in May 2018, marking a historic milestone as the first preventive therapy specifically designed for migraine (69). This was rapidly followed by the approval of fremanezumab (Ajovy) in September 2018, galcanezumab (Emgality) in September 2018, and eptinezumab (Vyepti) in February 2020, establishing a comprehensive therapeutic class within just 2 years (70).

In recent years, monoclonal anti-CGRP antibodies and CGRP receptor antagonists have demonstrated satisfactory efficacy in both preventive and acute treatments (71–73). A comprehensive 2024 systematic review examining real-world evidence and clinical trials confirmed that anti-CGRP monoclonal antibodies demonstrate substantial effectiveness, with more than 50% of patients achieving at least a 50% reduction in monthly headache days, while maintaining favorable tolerability profiles (74). Long-term studies through 2024 continue to support ongoing safety and efficacy for all four monoclonal antibodies, building evidence for earlier access to CGRP treatment as they increase quality of life and reduce monthly migraine days while being better tolerated than non-specific migraine preventative therapies (74). Extensive real-world evidence from multiple large-scale registries and cohort studies, along with numerous randomized controlled trials with extended follow-up periods, have demonstrated favorable long-term safety (75–77).

The clinical success of CGRP-targeted therapies has been reflected in evolving treatment guidelines. Major headache societies, including the American Headache Society, increasingly recognize these agents as cornerstone treatments for migraine prevention, with recent guidelines (78) supporting their consideration earlier in the treatment pathway for appropriate patients. This shift acknowledges both their substantial efficacy and favorable tolerability profile compared to many traditional preventive medications.

In addition to monoclonal antibodies, small-molecule CGRP receptor antagonists (gepants) such as rimegepant, ubrogepant, and atogepant have expanded treatment options for both acute and preventive therapy. These oral agents offer additional flexibility in dosing and route of administration while maintaining comparable safety profiles, with long-term data supporting good tolerability and no significant hepatotoxicity signals (79).

PACAP also demonstrates significant potential as a key therapeutic target. Although AMG 301, a monoclonal antibody targeting the primary PACAP receptor, showed no advantage over placebo (80), Lu AG09222, an anti-PACAP monoclonal antibody, exhibited notable therapeutic efficacy in randomized controlled trials (81, 82). Similarly, the success of PACAP monoclonal antibodies offers a compelling rationale for revisiting the potential therapeutic effects of substance P (SP). The early failures of neurokinin-1 receptor antagonists do not appear to hinder the future development of monoclonal antibody-based therapies (83).

#### 4.3.3 Genetics of migraine

Ongoing advancements in genetic research have provided valuable insights into the molecular mechanisms underlying the pathophysiology of migraine. Rare monogenic migraine, such as familial hemiplegic migraine, are caused by mutations in genes that encode ion channels and transporters (84). The three primary genes involved are CACNA1A, which encodes Cav2.1 channels; ATP1A2, which encodes the α2 Na+/K + -ATPase; and SCN1A, which encodes Nav1.1 channels. Mutations in these genes lead to the three subtypes of familial hemiplegic migraine (85). GWAS are a systematic approach to detecting genetic variations across the entire genome, identifying genetic variants associated with diseases or traits through differences in allele frequencies between groups (86). For common polygenic migraine, the application of GWAS has played a crucial role. To date, dozens of potential pathogenic genes have been identified. These genes are thought to be involved in the encoding of various transcription factors, ion channels, transmembrane transporters, cell adhesion proteins, receptor proteins, and enzymes. They are implicated in multiple mechanisms, including neuronal plasticity, differentiation, development, and metabolism of various cells, as well as immune responses (53, 84). In conclusion, identifying the key genetic factors underlying migraine will contribute to the further optimization of diagnosis and targeted therapies, while also providing new directions for future drug development.

#### 4.3.4 Trigeminovascular system and pain regulation networks in migraine

The onset of migraine is closely associated with the activation of the trigeminovascular system. The pain modulation network in migraine is a complex, multi-level system that involves multiple regions, including the trigeminal system, limbic system, brainstem, thalamus, and cortex, as well as their interactions. Pseudounipolar trigeminal afferent fibers, located in the trigeminal ganglion, innervate extracranial structures involved in pain perception, including the meninges and their associated vasculature. Upon activation or sensitization of the trigeminal nerve, a cascade of substances, including CGRP, PACAP-38, and SP, is released. This release triggers local inflammatory responses and vasodilation. Nociceptive signals transmitted by the trigeminal nerve reach the trigeminocervical complex and subsequently ascend to the thalamus. Within the thalamus, these complex peripheral sensory and nociceptive inputs are integrated and processed before being transmitted to the cortex. Here, they interact with cortical regions mediating higher functions such as vision, hearing, and motor control, forming intricate connections that ultimately contribute to the perception of migraine (87, 88). Additionally, neurons in the trigeminal cervical complex can directly connect with pain-regulating structures such as the hypothalamus and the locus coeruleus (89, 90). The brainstem plays a crucial role in the modulation of pain associated with migraine, influencing the pain perception through the modulation of trigeminal nerve signaling. Specifically, structures such as the medial reticular formation and regions surrounding the cerebral peduncle, are considered essential in the transmission of migraine-related signals (21). Brainstem activation during migraine attacks has been well-documented (29). Some researchers suggest that brainstem activation during migraine may exert an analgesic effect (21). Overactivation of the anterior hypothalamus is believed to be associated with the chronicity of migraine (91). The limbic system is also considered to play a significant role in the progression of migraine (92). Additionally, structures such as the basal ganglia (93) and cerebellum (94) are deeply involved in the transmission and interaction of migraine-related signals. Together, these structures form a complex pain modulation network.

#### 4.3.5 Neuroimmunology

For a long time, due to the autonomy of the immune system and the presence of the BBB, scholars generally believed that the immune system and the nervous system functioned independently. However, with the continuous advancement of knowledge, this traditional view was gradually overturned (95, 96). The bidirectional interplay between the immune and nervous systems has garnered substantial attention. Extensive research has underscored its crucial role in maintaining homeostasis, combating infections, and responding to injury, through diverse mechanisms such as the activation of glial cells, their transformation into neurotoxic or neuroprotective phenotypes, and the expression of chemokines and cytokines (95, 97). The discovery of the glymphatic system also provided a novel anatomical perspective. Distinct from the classical lymphatic system, the glymphatic system is a complex network of perivascular spaces containing large volumes of cerebrospinal fluid (CSF) and interstitial fluid (ISF), which surround cerebral blood vessels and communicate with the subarachnoid space (98). The direct connection between CSF and ISF with cervical lymphatics may serve as a direct pathway for the transmission of neuroimmune signals by modulating pro-inflammatory substances, CGRP, and related ion concentrations. Furthermore, neuroimmunology plays a critical role in the crosstalk among microglia, astrocytes, oligodendrocytes, and neurons (99–101). This involvement likely encompasses multiple mechanisms, including activation of the trigeminovascular system, central sensitization, and hyperalgesia. These processes are closely associated with various clinical manifestations, such as aura and photophobia, and are deeply implicated in the initiation, persistence, and chronification of migraine.

#### 4.3.6 Aura

Migraine aura is defined by transient focal neurological disturbances, typically involving visual, sensory, language, or motor symptoms (22). Clinically, approximately 30% of patients report experiencing aura, which usually lasts between 5 min and 1 h, with visual phenomena being the most prevalent (102, 103). The underlying mechanisms of migraine aura remain incompletely understood; however, current prevailing theories suggest that CSD and neuroinflammatory processes play critical roles. CSD is an abnormal electrical activity phenomenon that occurs in cortical neurons and glial cells. Initially, it involves massive depolarization of these cells, accompanied by significant local hyperemia. Subsequently, CSD propagates slowly at a rate of approximately 3–4 mm/min and is followed by a prolonged phase of reduced cerebral blood flow that can last for up to an hour or even longer (104, 105). During CSD, significant alterations occur in cellular morphology and in the intracellular and extracellular concentrations of ions such as sodium, potassium, and calcium. This process is accompanied by the release of various neurotransmitters, inflammatory mediators, and vasoactive substances. The resulting vasodilation, plasma protein extravasation, microglial activation, and tissue edema may further contribute to neuroinflammation and trigeminovascular system activation, ultimately triggering migraine (102, 106).

#### 4.3.7 Comorbidities

As a complex neurological disorder, migraine is intricately associated with a diverse range of comorbid diseases and has attracted considerable research attention. Current discussions on migraine comorbidities encompass multiple disease categories, such as psychiatric, cardiovascular and cerebrovascular, metabolic, endocrine, immune, and gastrointestinal disorders (107–110). However, the mechanisms underlying comorbidities remain poorly understood. The academic community has explored several potential aspects of comorbid mechanisms: (a) Migraine may lead to other diseases. (b) Other diseases may lead to migraine. (c) Bidirectional causality, such as in the case of major depression (107). (d) Comorbidities related to medications and treatment factors. For instance, long-term medication use may lead to MOH and adverse drug effects can introduce new comorbidities or exacerbate existing ones, such as weight gain associated with tricyclic antidepressants (111, 112). (e) Shared genetic, environmental, and other etiological factors may underlie the coexistence of migraine with other conditions. For instance, alterations in neurotransmitters such as glutamate and GABA; CSD; and mutations in genes including CACNA1A, ATP1A2, and SCN1A have been shown to be highly correlated with both migraine and epilepsy (113). Inflammation, immune dysregulation, endocrine imbalances, and autonomic nervous system dysfunction have been demonstrated to play significant roles in comorbidities associated with migraine, such as obesity and menstrual migraine (110, 114). Additionally, functional brain network disruptions should not be overlooked, as numerous psychiatric comorbidities have been found to overlap with migraine in regions such as the prefrontal cortex, fusiform gyrus, and thalamocortical circuits, as revealed by advanced techniques like fMRI (115).

### 4.4 Current research

To further investigate the current focus of research within the field, we reviewed studies published from 2025 to the present. The findings indicate that a substantial number of clinical trials are ongoing, primarily evaluating the efficacy and safety of various pharmacological treatments (116, 117), while sustained interest remains in non-pharmacological interventions such as acupuncture and transcranial magnetic stimulation (118, 119). In addition, considerable attention has been devoted to specific populations such as women (120), associated symptoms including insomnia (121), and phenomena such as suicide rates (122). The genetic mechanisms underlying migraine have also attracted significant attention. A study on single nucleotide polymorphisms identified the IL1B -3953\u00B0C/T variant as a potential biomarker for migraine susceptibility (123). The association between familial hemiplegic migraine-related genes (CACNA1A, ATP1A2, and SCN1A) and both migraine and epilepsy (124), as well as the impact of polymorphisms in pain-related sodium channels SCN9A and SCN10A on migraine chronification (125), have been further elucidated. Focusing on the crucial role of the trigeminovascular system and pain modulation networks, detailed investigations into the pathophysiological mechanisms of rare cases associated with the trigeminovascular system have been conducted (126). The relationship between CGRP and glutamate has been explored in the context of peripheral and central sensitization in migraine (127). The significant role of tryptophan metabolism abnormalities and the kynurenine pathway in neuroregulation in pediatric migraine patients has been affirmed (128). The importance of aura, cortical spreading depression, and blood flow changes has been consistently emphasized through extensive clinical and basic research (129–131). In addition, advanced methodologies such as machine learning (132), radiomics (133), fMRI (134), and multi-omics analyses (135) have been widely applied. Notably, increasing attention has been directed toward obesity (136), the gut microbiota, and dietary factors (137–139), highlighting the potential role of the gut–brain axis in the pathophysiology of migraine.

### 4.5 Future perspectives

Based on our analysis, future research trends in this field are reasonably projected. Investigations into the diverse pathophysiological mechanisms of migraine are expected to continue, with particular emphasis on episodic migraine and its subtypes. Sustained focus on the CGRP system will aim to generate robust long-term data on the safety, efficacy, and tolerability of CGRP-targeted therapies, while fostering the development of novel agents within this pathway. Attention will also be directed toward related comorbidities and complications, accelerating the clinical translation of potential therapeutic targets and the integration of emerging technologies, all underpinned by rigorous methodological standards. The gut–brain axis is poised to receive increased scrutiny through comprehensive mechanistic evaluations. Efforts will persist in constructing and refining the evidence-based medicine framework, enhancing multidimensional evaluation models for both mainstream and emerging therapies. Simultaneously, the clinical evidence supporting non-pharmacological interventions—such as acupuncture and surgical approaches—will be fortified to facilitate their broader clinical adoption.

### 4.6 Limitations

This study has several limitations that must be clearly acknowledged. To ensure the quality of the research, only data from both WOS and PubMed databases were included, which may have resulted in the exclusion of some relevant studies. While databases such as Scopus and Cochrane could provide additional coverage, particularly for regional journals, conference proceedings, and systematic reviews, our choice of WOS and PubMed was justified by their complementary strengths in multidisciplinary citation analysis and biomedical indexing, the high quality and consistency of their metadata, their proven coverage of high-impact migraine research, and the reproducibility of our methodology. Additionally, restricting our analysis to English-language publications may have introduced language bias and potentially excluded valuable contributions published in other languages, particularly those from non-English-speaking regions where migraine research is actively conducted. The use of citation count as a selection criterion inevitably underestimates the contributions of recently published high-quality literature. While we have provided data on the open access status and funding sources of the included publications in the Supplementary material, our study does not quantitatively analyze the relationship between these factors and citation counts. The influence of broader societal, cultural, economic, and editorial factors on research productivity and impact, while undoubtedly significant, was beyond the descriptive bibliometric focus of this analysis. Future research employing specialized economic or sociological methodologies would be valuable to explore these complex dynamics in the migraine research field. The manual inclusion and exclusion of studies also introduced potential subjective biases. Furthermore, the interpretation of the data is unavoidably influenced by the authors’ perspectives. Additionally, literature published after December 31, 2024, was not included in this analysis.

## 5 Conclusion

In summary, this study represents the first bibliometric analysis to visualize the 200 most influential publications in migraine research, examining key aspects including journals, authors, countries/regions, institutions, keywords, and references. Based on these analyses, we summarized the current knowledge base, reasonably projected future trends, and provided an overview of prevailing research hotspots. We anticipate that researchers will continue to focus on the diverse pathophysiological mechanisms of migraine, with particular emphasis on episodic migraine subtypes and the critical role of the CGRP system. Through rigorous methodologies and the application of emerging technologies, efforts will persist to build and refine the evidence-based medicine framework, comprehensively evaluating the long-term safety, efficacy, and tolerability of therapies. Additionally, multidimensional evaluation models for both established and emerging treatments will be improved, alongside strengthening the clinical evidence base supporting non-pharmacological interventions.

### 5.1 Recommendations for future research in the field

- Enhance cross-regional collaboration by conducting large-scale, multicenter, double-blind randomized controlled trials to generate high-quality clinical evidence on the efficacy and safety of various treatment approaches.
- Improve the development of animal models for migraine and its comorbidities to better reflect clinical complexity and facilitate translational research.
- Promote the clinical translation of potential therapeutic targets, such as PACAP and substance P (SP), with particular emphasis on the development of monoclonal antibody-based therapies.
- Emphasize research on non-pharmacological interventions for migraine prevention and symptom relief, especially in reducing drug dosage or side effects, to support the evidence base for integrative, multimodal treatment strategies.
- Investigate therapies targeting the peripheral nervous system, and explore the theoretical foundations for interventions involving adjacent muscular, fascial, and peripheral nerve structures.
- Maintain sustained attention to comorbidities and associated symptoms, as these play a crucial role in disease burden, diagnosis, and treatment optimization.

## Data Availability

The original contributions presented in the study are included in the article/Supplementary material, further inquiries can be directed to the corresponding author/s.

## Footnotes

1
https://www.webofscience.com/wos/woscc/basic-search

2
https://pubmed.ncbi.nlm.nih.gov

3
https://jcr.clarivate.com/jcr/browse-journals
