# Supplementary material for: The 200 most influential publications in migraine research: a bibliometric mapping of the intellectual landscape
Source: Front Neurol. 2025 Dec 1;16:1711571. doi: 10.3389/fneur.2025.1711571 (PMC12702772; doi:10.3389/fneur.2025.1711571)
Supplement: Supplementary file 1 [file Data_Sheet_1.docx]

Supplementary Material

# Supplementary Data 1

Web of Science：

TS=(Migraine, Hemicrania) OR TS=(Migraine Disorders) OR TS=(Migraine Headaches) OR TS=(Migraine, Abdominal) OR TS=(Migraine Variants) OR TS=(Migraine, Acute Confusional) OR TS=(Migraine Disorders) OR TS=(Migraine Headache) OR TS=(Migraine Variant) OR TS=(Migraine Syndromes, Cervical) OR TS=(Migraine Syndrome, Cervical) OR TS=(Migraine) OR TS=(Migraines, Hemicrania) OR TS=(Migraines, Abdominal) OR TS=(Migraines, Acute Confusional) OR TS=(Migraines) OR TS=(Hemicrania Migraine) OR TS=(Hemicrania Migraines) OR TS=(Disorder, Migraine) OR TS=(Disorders, Migraine) OR TS=(Migraine Disorder) OR TS=(Headache, Migraine) OR TS=(Headaches, Migraine) OR TS=(Acute Confusional Migraine) OR TS=(Acute Confusional Migraines) OR TS=(Status Migrainosus) OR TS=(Variant, Migraine) OR TS=(Variants, Migraine) OR TS=(Sick Headache) OR TS=(Headache, Sick) OR TS=(Headaches, Sick) OR TS=(Sick Headaches) OR TS=(Abdominal Migraine) OR TS=(Abdominal Migraines) OR TS=(Cervical Migraine Syndrome) OR TS=(Cervical Migraine Syndromes).

PubMed：

("Migraine"[Mesh] OR Migraine[TIAB] OR Hemicrania[TIAB] OR "Migraine Disorders"[TIAB] OR "Migraine Headaches"[TIAB] OR "Migraine, Abdominal"[TIAB] OR "Migraine Variants"[TIAB] OR "Migraine, Acute Confusional"[TIAB] OR "Migraine Headache"[TIAB] OR "Migraine Variant"[TIAB] OR "Migraine Syndromes, Cervical"[TIAB] OR "Migraine Syndrome, Cervical"[TIAB] OR Migraines[TIAB] OR "Hemicrania Migraine"[TIAB] OR "Hemicrania Migraines"[TIAB] OR "Disorder, Migraine"[TIAB] OR "Disorders, Migraine"[TIAB] OR "Migraine Disorder"[TIAB] OR "Headache, Migraine"[TIAB] OR "Headaches, Migraine"[TIAB] OR "Acute Confusional Migraine"[TIAB] OR "Acute Confusional Migraines"[TIAB] OR "Status Migrainosus"[TIAB] OR "Variant, Migraine"[TIAB] OR "Variants, Migraine"[TIAB] OR "Sick Headache"[TIAB] OR "Headache, Sick"[TIAB] OR "Headaches, Sick"[TIAB] OR "Sick Headaches"[TIAB] OR "Abdominal Migraine"[TIAB] OR "Abdominal Migraines"[TIAB] OR "Cervical Migraine Syndrome"[TIAB] OR "Cervical Migraine Syndromes"[TIAB])
